# Learning emotional dialects: A British population study of cross-cultural communication

**DOI:** 10.1177/03010066231204180

**Published:** 2023-10-05

**Authors:** Myron Tsikandilakis, Persefoni Bali, Renzo C. Lanfranco, Leonie Kausel, Zhaoliang Yu, Gonzalo Boncompte, Alexandros-Konstantinos Karlis, Alkadi Alshammari, Ruiyi Li, Alison Milbank, Michael Burdett, Pierre-Alexis Mével, Christopher Madan, Jan Derrfuss

**Affiliations:** 6123 University of Nottingham, UK; University of Edinburgh, UK; 27106 Karolinska Institutet, Sweden; Universidad Diego Portales, Chile; Wuhan University, China; 37580 National University of Singapore, Singapore; Pontificia Universidad Católica de Chile, Chile; 68993 National and Kapodistrian University of Athens, Greece; 37850 King Saud University, Saudi Arabia; Sungkyunkwan University, Korea; 6123 University of Nottingham, UK

**Keywords:** learning, culture, dialects, emotion, morphing

## Abstract

The aim of the current research was to explore whether we can improve the recognition of cross-cultural freely-expressed emotional faces in British participants. We tested several methods for improving the recognition of freely-expressed emotional faces, such as different methods for presenting other-culture expressions of emotion from individuals from Chile, New Zealand and Singapore in two experimental stages. In the first experimental stage, in phase one, participants were asked to identify the emotion of cross-cultural freely-expressed faces. In the second phase, different cohorts were presented with interactive side-by-side, back-to-back and dynamic morphing of cross-cultural freely-expressed emotional faces, and control conditions. In the final phase, we repeated phase one using novel stimuli. We found that all non-control conditions led to recognition improvements. Morphing was the most effective condition for improving the recognition of cross-cultural emotional faces. In the second experimental stage, we presented morphing to different cohorts including own-to-other and other-to-own freely-expressed cross-cultural emotional faces and neutral-to-emotional and emotional-to-neutral other-culture freely-expressed emotional faces. All conditions led to recognition improvements and the presentation of freely-expressed own-to-other cultural-emotional faces provided the most effective learning. These findings suggest that training can improve the recognition of cross-cultural freely-expressed emotional expressions.

Financial opportunities, favourable immigration policies and international academic accessibility have all contributed to the plurality of our cross-cultural societies. Easy access to social media that allows for professional collaborations and social interactions at our work or domestic environment have also contributed to contact with individuals from other cultures. We frequently communicate with people from other cultures, and it is therefore only sensible to ask how we might better understand the emotional expressions of those in our culturally diverse environments because it is fundamental for effective communication.

One psychological model related to cross-cultural emotional communication suggests that there are universals in the expression of emotion (Ekman & Friesen, 1971). These universals refer to prototypical expressions of emotion that are expressed and understood in every society. This is suggested to occur because these have evolutionary value. They are a fundamental communication currency for all societies because they provide the means to express and recognise important emotional states (Ekman, 2004). These emotions are communicated via facial expressions, frequently referred to as basic universal emotional expressions (Biehl et al., 1997; Ekman & Cordaro, 2011; Ortony & Turner, 1990; see also Keltner et al., 2019). These include anger, fear, disgust, happiness, sadness, surprise and neutral facial expressions. The components of these facial expressions are called Action Units (AUs; Ekman & Friesen, 1978). Combinations of these AUs, such as specific mouth and eye movements, are suggested to form cross-culturally recognizable facial expressions of emotion (Ekman, 2004; Essa & Pentland, 1997; but see also Solomon & Stone, 2002).

Related research suggests that prototypical emotional expressions, such as anger, fear, disgust, happiness, sadness, surprise and neutral expressions, are a universal language of human communication (Russell et al., 2003; Siedlecka & Denson, 2019). These universal facial expressions can also be expressed with specific and distinguishable AUs in different cultures forming culture-specific dialects of emotion (see Elfenbein et al., 2007; Elfenbein, 2013, 2015; Gendron et al., 2015).

This is suggested to occur because different cultures have specific display, such as expressive and decoding emotional recognition, rules that stem from the specific evolutionary trajectory of each culture. These are, therefore, better recognizable by own-culture members (Coan & Gottman, 2007; Crivelli & Fridlund, 2019; Elfenbein & Ambady, 2002a, 2002b, 2003; Hwang & Matsumoto, 2015). This phenomenon is termed the own-culture emotional recognition advantage (Elfenbein & Ambady, 2002a, 2002b). It refers to the ability to recognise dialects of emotion from one's own culture more accurately than dialects of emotion from other cultures (Hess et al., 2016; but see also Matsumoto, 2002).

One scientific debate relating to the own-culture recognition advantage is whether own-culture faces have increased evolutionary communication value and can be recognised without conscious awareness. Previously relevant research involved evidence that conscious awareness is not required for the processing of own-culture facial-emotional dialects. Relevant studies to date have utilised methods such as flash suppression; that is, the brief simultaneous presentation of target and noise stimuli. They have also utilised backward masking; that is, the suppression of a brief target stimulus with a noise image. The provided evidence pointed towards the subliminal processing of own-culture emotional faces (Chiao et al., 2008; Cunningham et al., 2004; Eberhardt et al., 2004; Smith et al., 2008; Zebrowitz et al., 2008; see also Brooks et al., 2012; Kawakami & Yoshida, 2019; van der Ploeg et al., 2017).

We have elsewhere highlighted the methodological limitations of previous findings (Tsikandilakis, Bali, et al., 2019b, 2020; Tsikandilakis, Bali, Haralabopoulos, et al. 2020; Tsikandilakis, Yu, et al. 2021; Tsikandilakis, Bali, Karlis, et al. 2023a, 2023b). These include the use of biased metrics for the definition of unconsciousness (i.e., hit rates). These include the assessment of unconsciousness using statistical procedures that confound lack of evidence for the alternative hypothesis with evidence for the null. These also include the lack of separate statistical analyses for trials in which participants reported hit responses, such as seeing a face that was presented, and miss responses, such as not seeing a face that was presented (Tsikandilakis, Bali, Karlis, et al. 2023a).

In two previous publications, we assessed whether own-culture faces can be processed without awareness, and we implemented a novel methodology for unconsciousness (Tsikandilakis, Bali, et al., 2019; Tsikandilakis, Yu, et al. 2021). We employed non-parametric receiver operating characteristics (Stanislaw & Todorov, 1999; Zhang & Mueller, 2005; see also Dienes, 2021), Bayesian analyses for unconscious perception (Dienes, 2014, 2015, 2016, 2019) and hits-versus-miss analyses for assessing participant responses (Lynn & Barrett, 2014; Pessoa, 2005; Pessoa & Adolphs, 2010; Pessoa et al., 2005; Sergeev et al., 2018; Wixted, 2020).

We showed that when own-culture emotional faces were presented with backward masking for 33.33 ms and assessed with unbiased criteria for signal detection and discrimination performance, participants could correctly identify a presented face only when conscious perception was involved. These findings were replicated in our research in the past five years (Tsikandilakis & Chapman, 2018; Tsikandilakis et al., 2018, Tsikandilakis, Bali, et al., 2019, Tsikandilakis, Bali, et al., 2019, 2020, 2020b; Tsikandilakis, Yu, et al. 2021a; Tsikandilakis, Bali, et al. 2021; Tsikandilakis, Leong, et al., 2021; Tsikandilakis, Bali, Yu, et al., 2023; Tsikandilakis, Bali, Karlis, et al. 2023a, 2023b).

These findings provided us with a methodological starting point for the present research. In this present research project, we aimed to explore whether we could implement interventions to improve cross-cultural emotional communication. Based on the findings of our own research group, the implementation of these interventions was designed to involve conscious perception of cross-cultural freely-expressed emotional dialects of emotion, as opposed to unconscious or implicit tasks (see van der Ploeg et al., 2017). In the current research project, we attempted to explore, using explicit and interactive methods involving face presentations, ways to improve the recognition of other-culture emotional faces in British participants.

As it has become increasingly acknowledged and criticised, most psychological studies are conducted within a constrained demographic sample relative to the overall world population (see Henrich et al., 2010). As our world and daily interactions become more internationalised, we need to be more culturally inclusive, and this involves studying and developing better approaches for understanding other cultures. As our society moves forward, we need to work to remove cultural barriers and instead seek to improve cross-cultural communication. For example, Barrett (2020) highlights how cognitive science is well situated as a field to bridge across cultures, with research topics occurring at different scales, across societal cognition, interpersonal cognition, and individual cognition. Emotions and communications are at the heart of this, playing a critical role in how we understand and relate to others (e.g., theory of mind). The present work represents our contribution to improving cross-cultural communication, an important aspect of everyday cognition as globalisation continues.

Due to the lack of previous research initiatives for improving cross-cultural emotional recognition, in the current research project we implemented a plethora of methods ranging from basic, such as simple presentation, to complex methods, such as dynamic morphing to explore how to improve cross-cultural emotional recognition (Calvo & D'Mello, 2010; Kask & Bull, 2009; Russell, 1994; Sutherland et al., 2017; see also Dawel et al., 2021). All methods of face presentation included the overt presentation of faces. They included participant well-being oriented interactive feedback (see Bond et al., 2021) and included catch-trial methods for providing reliably subjective unbiased responses for the recognition of emotion (Haralabopoulos et al., 2020).

Our aim in the current research project was to find the most effective method for improving the cross-cultural recognition of emotion. We aimed to test a sufficient number of explorative methods to be able to report results for an intervention that provided Bayesian evidence for levelling the own-culture recognition advantage meaning in this context a method that would provide evidence for the likelihood that the recognition of own and other-culture faces was statistically proximate. We had two additional aims. Firstly, we wanted to explore whether we could provide replications of the own-culture recognition advantage. Secondly, we wanted to assess what – if any – is the role of racial familiarity in the outcomes of the current research.

Our hypotheses were that contemporary methods that provide highly discernible visual information, such as dynamic morphing, would be most effective in improving the cross-cultural recognition of faces. We hypothesised that this effect should involve the presentation of both other and own-culture emotional faces because the latter could function as a comparison and reference point for recognition improvements (Barrett, 2020; Gendron et al., 2015). Finally, we hypothesised that our finding would involve replications of the own-culture emotional recognition advantage (Elfenbein & Ambady, 2002a, 2002b, 2003; Elfenbein, 2013, 2015) and evidence that emotional expression and not racial familiarity was the contributing variable for the reported outcomes (Amihai et al., 2011; Laukka & Elfenbein, 2021).

## Experimental Stage One

### Aims

The main aim of this stage was to explore the most effective method for improving the recognition of faces showing freely-expressed cross-cultural dialects of emotion in a British participant sample. Further aims of this stage involved exploring whether we could provide a replication of the own-culture emotional recognition advantage including higher confidence ratings for own-culture emotional recognition and emotional familiarity.

### Participants

A power calculation based on medium sizes indicated that 180 participants were required for this stage (*P*_(1−β)_ ≥ .9; *p* ≤ .05; η^2^_p _≥ .06; Faul et al., 2009; *P* (H_0_) ≥ .9; *B* < .33; η^2^_p_ [0,  < .001]; Kelter, 2021). A total of 199 participants (97 female) volunteered to participate in this study. All participants reported normal or corrected-to-normal vision. The inclusion criteria for the current study were having been born in Britain, having attended primary, secondary, and higher education in Britain and in the English language, having previously resided only and currently residing permanently in Britain, and characterising themselves as part of the British culture (Yes/No). Participants were additionally screened with the Somatic and Psychological Health Report Questionnaire (SPHRQ; Hickie et al., 2001) and an online Alexithymia-Emotional Blindness Questionnaire (Alexithymia, 2021). Data from ten participants were excluded because they failed to attend the second and third phases of the experiment. Data from five participants were excluded due to SPRHQ scores that indicated a possible psychiatric diagnosis. Data from two participants were excluded due to scores that indicated possible traits for alexithymia. Data from two participants were excluded due to having a joint nationality. The final sample consisted of 180 participants (90 female) with mean age 24.44 years (SD = 2.01; see Table 1).

**Table 1.** Participant characteristics for experimental Stage One.

Method of presentation	*n* (female)	Age mean (SD)	CDQ mean (SD)					ERQ mean (SD)	
			PD	IND	MAS	U-A	LTO	CR	ES
No intervention	30 (15)	24.67 (2.01)	44.6 (4.38)	62.1 (3.03)	45.47 (4.02)	68.93 (2.99)	53.63 (2.2)	25.03 (3.05)	8.23 (1.43)
Shape assessment	30 (15)	25.71 (1.73)	44.87 (4.64)	63.83 (2.89)	45.17 (3.71)	69.33 (3.04)	53.27 (2.74)	24.3 (3.08)	8.13 (1.43)
Mere exposure	30 (15)	24.51 (1.55)	44.86 (4.58)	63.13 (3.19)	46.71 (3.68)	68.17 (2.96)	53.73 (2.79)	24.21 (2.78)	7.97 (1.54)
Side-by-side	30 (15)	26.03 (2.07)	43.6 (4.62)	64.53 (3.74)	46.13 (3.99)	69.07 (3.31)	53.27 (2.5)	24.73 (2.99)	8.43 (1.45)
Back-to-back	30 (15)	25.76 (2.08)	46.37 (3.85)	63.17 (3.52)	45.48 (3.73)	69.63 (3.64)	54.13 (2.64)	25.02 (3.12)	8.27 (1.57)
Morphing	30 (15)	25.92 (2.65)	44.97 (4.13)	63.87 (3.89)	46.67 (3.44)	69.27 (3.3)	54.14 (2.22)	25.01 (7.61)	8.37 (1.19)
Bayes factor, ANOVA and effect sizes for each category									
*p*-value (B factor)			.24 (1.78)	.12 (2.14)	.49 (.93)	.62 (.71)	.63 (.69)	.79 (.43)	.84 (.39)
η^2^_p_			.04	.05	.03	.02	.02	.01	.01

This table includes participant *n* and age. It also includes mean and standard deviation percentiles for the Hofstede Cultural Dimensions Questionnaire (CDQ) with scores for power distance (PD), individualism (IND), masculinity (MAS), uncertainty-avoidance (U-A) and long-term orientation per country of origin (LTO; see Hofstede, 2003). It also includes scores for the emotional regulation questionnaire (ERQ) with scores for cognitive re-appraisal (CR) and emotional suppression (ES) per cohort. In the bottom part of the table, we present comparisons per cohort using both ANOVA and Bayesian analysis. Bayesian analysis was performed using the Dienes calculator with *B* < .33 signifying evidence for the null, .33 < *B* < 3 signifying anecdotal evidence and *B* > 3 signifying evidence for the alternate hypothesis (Dienes, 2016). Partial eta-squared scores for every analysis are also included in the bottom row. Asterisks (*) would indicate scores that are significantly different at *p* ≤ .01 to at least one other item of the same category; no reported instances. See also https://osf.io/3z97s/ and https://osf.io/cdvhz/ and https://osf.io/syvf9/. These outcomes suggested that cultural and emotionality-related factors, relating potentially to individual differences or racial differences (see Cohen, 2009), would not significantly impact the comparisons among the cohorts/methods of learning cross-cultural dialects of emotions and their control conditions.

After the initial screening processes, participants were asked to complete the Hofstede Cultural Dimensions Questionnaire (Hofstede, 2003) and the Emotional Regulation Questionnaire (Gross & John, 2003). Subsequently, the participants were randomly allocated to cohorts depending on the type of method that was used during phase two (see Table 1). Our aim for implementing these assessments was to discover, with traditional frequentist and Bayesian analyses, whether differences between cohorts could influence the results in subsequent stages due to differences in cultural and/or emotional sensitivity characteristics. All participants gave informed consent to participate in this study and for their data to be used for further research purposes. The experiment was approved by the School of Psychology, University of Nottingham.

### Procedure

All stimuli were presented on a 60 Hz HD monitor. The presentation was programmed in the coder and builder components of PsychoPy (Peirce, 2007). The stimuli were created and thoroughly assessed, rated and validated for emotional characteristics in previous international collaborations funded by Universitas 21 (see Tsikandilakis, Kausel, et al., 2019; pp. 6–8; Tsikandilakis, Yu, et al. 2021; pp. 6–9). They included faces from Britain, Chile, New Zealand and Singapore showing freely-expressed emotional expressions of anger, disgust, fear, sadness, surprise, happiness and neutral expressions.

The freely-expressed faces were photographed in each country of interest by researchers in local universities (i.e., University of Nottingham, National University of Singapore, The University of Auckland, New Zealand, and the Pontificia Universidad Católica de Chile). The inclusion criteria for the actors were having been born in country of interest, having attended primary, secondary, and higher education in the country of interest and in the language of the country of interest, having previously resided only and currently residing permanently in the country of interest, and characterising themselves as part of the culture of the country of interest (Yes/No). The freely expressed photographs were taken while participants were asked to freely express emotions as they would in interactions with people from the same country of origin (anger, disgust, fear, sadness, surprise and happiness) in three levels (mild, moderate and intense) and neutral expressions. A minimum of three photos per emotional expression were taken for each actor. The resulting stimuli were sorted and labelled. We included mid intensity freely expressed faces in the experiments, to control for possible effects relating to very subtle (low-intensity) or exaggerated (high-intensity) emotional expressiveness and approximate normative and realistic cultural responses (see Tsikandilakis, Yu, et al. 2021a). All mid-intensity freely expressed faces that met the cultural background and gender criteria in Noldus 9.1 (*n* = 350) and were confirmed as expressing freely-expressed emotions in a pilot study by members of the same country of origin who also fit the aforementioned inclusion criteria were included in the experimental stages (for a full review of the selection process, see Tsikandilakis, Kausel, et al., 2019; pp. 5–12).

Seventy pattern blurs including different shades of grey fillings were created in MATLAB using random pixel permutation and included in the dataset as catch trials to assess participant attention. The facial stimuli were adjusted for interpupillary distance. All stimuli were transformed to grey scale and resized to a 1024 × 768 pixels resolution. Their average luminance was averaged using SHINE, MATLAB. The stimuli were spatially aligned and placed in a white background crop (height: 6 cm, width: 4 cm; Tsikandilakis et al., 2020a, 2020b; Tsikandilakis, Leong, et al., 2021; see also https://osf.io/3z97s/ and https://osf.io/cdvhz/ and https://osf.io/syvf9/). All participants were presented with the same facial stimuli. No facial stimuli were repeated within-participants between different phases.

### Procedure: Phase One

**Figure 1.**
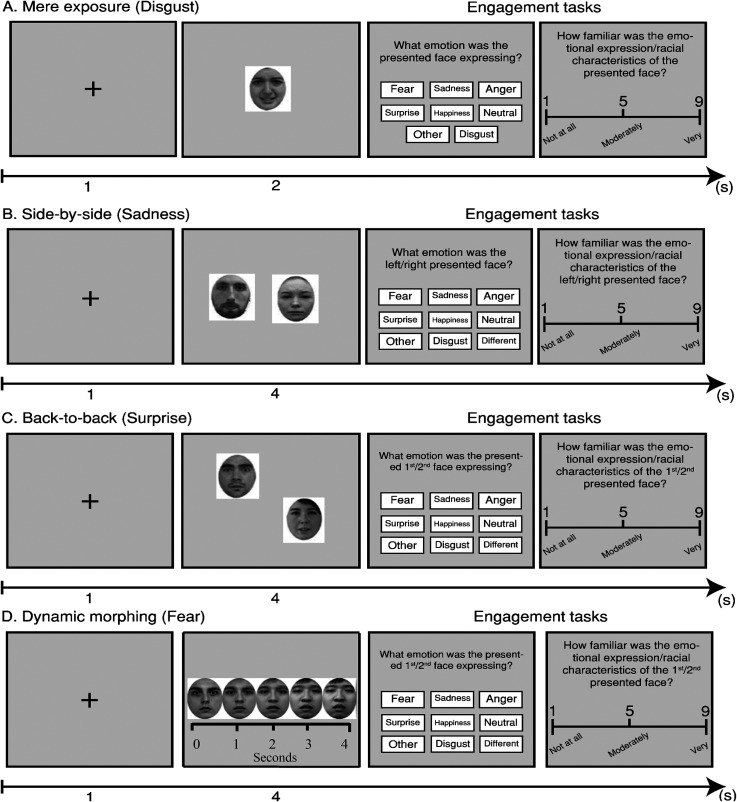
Face presentation conditions and engagement tasks used in Stage One, phase two: (A) Mere exposure. (B) Side-by-side. (C) Back-to-back. (D) Dynamic morphing. Dynamic morphing is shown as a function of time in seconds. Examples of methods presented here can be found in https://osf.io/3z97s/ and https://osf.io/cdvhz/ and https://osf.io/syvf9/.

The first experimental stage included three phases. In phase one all cohorts were presented with a fixation cross for 1 s. After the fixation cross, a single freely-expressed face from each country of origin (Britain, Chile, New Zealand and Singapore) showing anger or disgust or fear or sadness or surprise or happiness or a neutral expression, or a pattern blur, was presented for 2 s (*n*_trials overall_ = 350). Each emotion, including neutral faces, involved the presentation of five male and five female actors from each country of origin. After the presentation, a 1-s blank screen interval was shown. After the interval, participants were asked to choose from a list the emotion the face was expressing from anger or fear or disgust or surprise or sadness or happiness or neutral or non-facial or other using the mouse. Participants used the mouse to make their recognition selection from eight textboxes with order of positioning randomised in each trial. This question was always sequenced by a Likert scale consisting of a confidence rating for their choice from 1 (not at all) to 5 (moderately) to 9 (very). The participants were also asked to perform two more tasks. They were asked to rate how familiar they were with the emotional expression of the presented face and how familiar they were with the racial characteristics of the presented face from 1 (not at all) to 5 (moderately) to 9 (very). In case of the selection of a non-facial stimulus option, participants were asked how familiar they were with the presented shape and the shade of filling of the presented stimulus. The emotional recognition and confidence for emotional recognition tasks were always presented in sequence. The emotional recognition and confidence, and the emotional and racial familiarity tasks were randomised in each trial (see Figure 1).

### Procedure: Phase Two

Phase two took place one week after phase one on the same day and timeslot as phase one. It included six between-subjects conditions. Thirty participants were randomly allocated in each condition. The conditions were no intervention (**NI**), shape assessment (**SA**), mere exposure (**ME**), side-by-side (**SBS**) back-to-back (**BTB**) and dynamic morphing (**DM**). In the **NI** condition, participants simply had to confirm on-line that they were aware of the timeslot allocated and that they were still eligible for phase three. For the **SA** condition the experiment started with a fixation cross for 1 s. After the fixation cross, a single triangle or pentagon or hexagon or rectangle or circle or square or rhombus or oval shape was shown for 2 s. The shapes were randomly filled with different shades of grey (*n*_trials overall_ = 350). After each presentation, a blank screen was shown for 1 s. Participants were then asked to answer the kind of shape they saw from a list, including “other.” Participants used the mouse to make their recognition selection in each trial from eight two-by-five (2 × 5) cm textboxes with order of positioning randomised in each trial. This question was always followed by a Likert scale on how confident they were for their answer ranging from 1 (not at all) to 5 (moderately) to 9 (very). Participants were also asked to rate from 1 (not at all) to 5 (moderately) to 9 (very) how familiar they were with the shape and the presented grey filling in each presented shape, order randomised. After the answers, we used conditional branching. In case of a correct recognition answer, an on-screen message stated “Congratulations, your shape recognition response was correct.” In case of an incorrect recognition response, an on-screen message stated, “I am sorry, your shape recognition response was not correct.” The message lasted for 2 s, then an on-screen message stated “The correct answer was x” for 2 s (e.g., square or circle or oval) and the correct shape was shown again for 2 s. A blank screen interval period for 1 s was presented before the next trial (see Figure 1).

For the **ME** condition the experiment started with a fixation cross for 1 s. After the fixation cross, a single face was presented at fixation. The pool of faces included 70 own-culture (British), 70 Chilean, 70 New Zealand and 70 Singaporean faces, and 70 pattern blurs with order randomised. The faces included ten different angry or disgusted or fearful or sad or surprised or happy or a neutral expression from each culture for 2 s. Seventy pattern blurs were also presented as catch trials (*n*_trials overall_ = 350). After the presentation, a blank screen was shown for 1 s. Participants were then asked what emotion the face was expressing, including choices for “other” and “non-facial.” Participants used the mouse to make their recognition selection from eight two-by-five (2 × 5) cm textboxes with order of positioning randomised in each trial. This question was always sequenced by a Likert scale on how confident they were for their choice ranging from 1 (not at all) to 5 (moderately) to 9 (very). Participants were also asked to rate the presented face for emotional familiarity and racial familiarity from 1 (not at all) to 5 (moderately) to 9 (very). After the answers, we used conditional branching. In case of a correct recognition answer an on-screen message stated “Congratulations, your emotional recognition response was correct” or, in case of an incorrect response, “I am sorry, your emotional recognition response was not correct” for 2 s, followed by the on-screen message “The correct answer was x” for 2 s (e.g., fear or anger or disgust). Subsequently, the face was shown again for 2 s. A blank screen was presented for 1 s before the next trial (see Figure 1).

For the **SBS** condition, the experiment started with a fixation cross for 1 s. After the fixation cross, an own-culture emotional face and an other-culture emotional face were presented next to each other with order randomised. The two faces always expressed the same freely-expressed emotion. The faces included 70 own-culture, 210 other-culture faces and 70 pattern blurs with order randomised. The faces included ten different angry, disgusted, fearful, sad, surprised, happy or neutral expressions from each culture. The presentation lasted for 4 s. Thirty-five pairs of pattern blurs were also presented side-by-side as catch trials for assessing participant attention for 4 s (*n*_trials overall post-stimulus overlap_ = 175). After the presentation, a blank screen was shown for 1 s. Participants were then asked to answer what emotion the faces were expressing, including “other,” “different” and also including “non-facial.” Participants used the mouse to make their recognition selection from nine textboxes with order of positioning randomised in each trial. This question was always sequenced by a Likert scale on how confident they were for their choice ranging from 1 (not at all) to 5 (moderately) to 9 (very). Participants were also asked to rate in pseudorandomised order (resulting in 50% responses for) either the left or right face for emotional familiarity and racial familiarity from 1 (not at all) to 5 (moderately) to 9 (very). After this task, we used conditional branching. In case of a correct recognition answer, an on-screen message stated “Congratulations, your emotional recognition response was correct” or, in case of an incorrect answer, “I am sorry, your emotional recognition response was not correct” for 2 s. Then an on-screen message stated “The correct answer was x” for 2 s (e.g., fear or anger or disgust) and the side-by-side faces were shown again for 4 s. A blank screen was presented for 1 s before the next trial (see Figure 1).

For the **BTB** condition, the experiment started with a fixation cross for 1 s. After the fixation cross, an own-culture emotional face and a different-culture emotional face were presented after one-another (one first and then the other in a pseudo-backward-masking fashion) with order randomised. The two faces always expressed the same freely-expressed emotion. The presentation lasted for 4 s divided equally between the two presented faces. The faces included 70 own-culture, 210 other-culture faces and 70 pattern blurs with order randomised. The faces included ten different angry, disgusted, fearful, sad, surprised, happy, neutral expressions from each culture. Thirty-five pairs of pattern blurs were also presented back-to-back as catch trials (*n*_trials overall post-stimulus overlap_ = 175). After the presentation, a blank screen was shown for 1 s. Participants were then asked to answer what emotion the faces were expressing, including “other,” “different” and “non-facial.” Participants used the mouse to make their recognition selection from nine textboxes with order of positioning randomised in each trial. This question was always sequenced by a Likert scale on how confident they were for their choice from 1 (not at all) to 5 (moderately) to 9 (very). Participants were also asked to rate in pseudorandomised order (resulting in 50% responses for) either the first or second face for emotional familiarity and racial familiarity from 1 (not at all) to 5 (moderately) to 9 (very). After this task, we used conditional branching. In case of a correct recognition answer, an on-screen message stated “Congratulations, your recognition answer was correct” or, in case of an incorrect response, “I am sorry, your recognition answer was not correct” for 2 s. Then, an on-screen message stated “The correct answer was x” for 2 s (e.g., fear or anger or disgust) and the back-to-back faces were shown again for 4 s. A blank screen interval period for 1 s was presented before the next trial (see Figure 1).

For the **DM** condition the experiment started with a fixation cross for 1 s. After the fixation cross, an own-culture emotional face was morphed into an other-culture face or vice versa. The two faces always expressed the same freely-expressed emotion. The morphing lasted for 4 s. The refresh rate for dynamic morphing was set at 60 Hz (16.67 ms) and the resolution of the morphed images was set at 1024 × 768 pixels using Abrosoft Fantamorph Pro (for further morphing illustrations, parameters and code, see https://osf.io/syvf9/). The faces included 70 own-culture, 210 other-culture faces and 70 pattern blurs with order randomised. The faces included ten angry, disgusted, fearful, sad, surprised, happy or neutral expressions from each culture. Thirty-five pairs of pattern blurs were also presented in morph as catch trials (*n*_trials overall post-stimulus overlap_ = 175). After the presentation, a blank screen was shown for 1 s. Participants were then asked to answer what emotion the faces were expressing, including “other,” “different” and “non-facial.” Participants used the mouse to make their recognition selection from nine textboxes with order of positioning randomised in each trial. This question was always sequenced by a Likert scale on how confident they were for their choice ranging from 1 (not at all) to 5 (moderately) to 9 (very). Participants were also asked to rate in pseudorandomised order (resulting in 50% responses for) either the first or second face for emotional familiarity and racial familiarity from 1 (not at all) to 5 (moderately) to 9 (very). After this task, we used conditional branching. In case of a correct recognition answer, an on-screen message stated “Congratulations, your recognition answer was correct” or, in case of an incorrect answer, “I am sorry, your recognition answer was not correct” for 2 s. Then an on-screen message stated “The correct answer was x” for 2 s (e.g., fear or anger or disgust) and the morphing sequence was shown again for 4 s. A blank screen for 1 s was presented before the next trial (see Figure 1). No actor identity was repeated during any of the aforementioned conditions, within or between trials. The gender of the presented actors was randomised for own and other-culture faces in all conditions (see Figure 1).

### Procedures: Phase Three

Phase three took place one week after phase two and on the same day and timeslot as phase two. Phase three was a replication of phase one. All cohorts were presented with a fixation cross for 1 s. After the fixation cross, a single novel freely-expressed face showing anger or disgust or fear or sadness or surprise or happiness or a neutral expression was presented for 2 s from each culture. Seventy pattern blurs were also presented as catch trials (*n*_trials overall_ = 350). The engagement tasks were identical to phase one.

### Results: Phase One

To explore the emotional recognition outcomes in phase one, an analysis of variance with Independent Variables Cohort Group (No Intervention vs. Shape Assessment vs. Mere Exposure vs. Side-by-Side vs. Back-to-Back vs. Dynamic Morphing) and Actor Origin (Britain vs. Chile vs. New Zealand vs. Singapore) and Facial Emotion (Anger vs. Fear vs. Disgust vs. Sadness vs. Surprise vs. Happiness vs. Neutral) was run. The resulting model provided evidence for significant differences for Actor Origin (F (3, 87) = 859.27; *p* < .001; η^2^_p _= .97; SE = .17; *B* = +∞). Bonferroni-corrected comparisons showed that British expressions were recognised significantly better than Chilean (*p* < .001; *d* = 1.56), New Zealand (*p* < .001; *d* = 1.54) and Singaporean expressions (*p* < .001; *d* = 2.1; see Table 2). These results provided support for the suggestion that there is an own-culture emotional recognition advantage (Elfenbein & Ambady, 2002a). The analyses also revealed significant differences for Facial Emotion (F (6, 174) = 244.22; *p* < .001; η^2^_p _= .89; SE = .21; *B* = +∞) and a significant Facial Emotion to Actor Origin interaction (F (18, 522) = 13.44; *p* < .001; η^2^_p _= .32; SE = .19; *B* = +∞; see Table 2). Critically, no other significant effects were reported including non-significance for emotional recognition differences between the six Cohort Groups and Bayesian evidence for proximate emotional recognition scores between the six Cohort Groups (F (5, 145) = .43; *p* = .82; η^2^_p _= .01; SE = .22; *B* = .13). These findings suggested that there were no initial emotional recognition differences between the included groups that would bias emotional recognition comparisons in subsequent phases (see Table 2: A1). Similar outcomes were reported for response confidence for emotional recognition. An important effect of Actor Origin was reported (F (3, 87) = 669.86; *p* < .001; η^2^_p _= .96; SE = .02; *B* = +∞). Bonferroni-corrected comparisons revealed that British expressions were rated with significantly higher confidence for emotional recognition than Chilean (*p* < .001; *d* = .78), New Zealand (*p* < .001; *d* = .95) and Singaporean expressions (*p* < .001; *d* = 1.33). No other comparisons or interaction were significant for response confidence for emotional recognition (see Table 2: A1–2).

**Table 2.** Participant outcomes for phase one.

Country	Anger	Fear	Disgust	Happiness	Sadness	Surprise	Neutral	Overall
A1. Mean (SD) Emotional recognition (%).								
Britain (NI)*	87.04 (5.84)	88.48 (5.78)	88.89 (6.55)	86.9 (6.47)	89.64 (6.61)	89.03 (5.91)	90.26 (4.99)	88.61 (6.02)
Britain (SA)*	89.68 (5.54)	84.8 (6.08)	87.93 (6.19)	87.17 (7.09)	88.61 (6.62)	86.93 (6.43)	89.68 (5.09)	87.83 (6.15)
Britain (ME)*	87.96 (5.99)	88.27 (6.45)	87.07 (6.57)	89.92 (5.54)	88.72 (7.48)	86.49 (6.4)	91.29 (4.72)	88.53 (6.16)
Britain (SBS)*	87.58 (7.43)	85.9 (6.02)	88.1 (6.13)	87.21 (6.7)	88.34 (6.9)	86.42 (5.31)	91.57 (5.31)	87.87 (6.26)
Britain (BTB)*	86.52 (7.15)	79.31 (6.82)	92.7 (5.93)	93.73 (5.73)	80.34 (5.99)	89.61 (6.45)	88.58 (6.57)	87.26 (6.38)
Britain (DM)*	88.37 (5.89)	87.25 (6.34)	88 (6.42)	88.02 (6.66)	88.46 (6.35)	87 (5.43)	90.65 (4.66)	88.25 (5.96)
Chile (NI)	76.12 (5.79)	76.53 (6.65)	76.49 (6.49)	75.71 (6.43)	77.66 (7.74)	78.73 (6.37)	86.14 (6.09)	78.2 (6.51)
Chile (SA)	78.62 (5.45)	77.35 (6.92)	77.39 (6.09)	78.35 (6.13)	76.39 (7.41)	77.15 (6.91)	88.17 (6.31)	79.06 (6.46)
Chile (ME)	76.19 (5.84)	75.5 (5.95)	77.22 (6.62)	76.91 (5.04)	76.56 (6.98)	76.67 (7.01)	88.13 (6.76)	78.17 (6.31)
Chile (SBS)	76.29 (5.06)	76.77 (6.63)	76.36 (6.33)	74.95 (7.19)	77.25 (5.39)	76.36 (5.64)	88.51 (6.07)	78.07 (6.04)
Chile (BTB)	86.52 (5.54)	74.16 (7.48)	85.49 (6.4)	84.46 (4.71)	83.43 (5.84)	69.01 (5.95)	97.85 (6.61)	82.99 (6.08)
Chile (DM)	77.19 (6.49)	77.02 (6.87)	77.77 (5.94)	76.75 (6.09)	77.02 (6.63)	76.69 (5.06)	87.52 (5.89)	78.57 (6.14)
N.Z. (NI)	76.7 (6.07)	76.67 (5.86)	77.35 (5.85)	79.45 (5.74)	77.9 (6.99)	78.52 (6.51)	88.03 (6.29)	79.23 (6.19)
N.Z. (SA)	76.8 (5.44)	78.45 (6.29)	76.7 (5.88)	75.4 (5.34)	77.77 (5.93)	77.49 (6.8)	86.69 (6.5)	78.47 (6.03)
N.Z. (ME)	77.94 (6.19)	78.69 (6.06)	76.39 (5.62)	78.76 (7.73)	76.77 (5.79)	78.52 (6.13)	87.38 (6.34)	79.21 (6.27)
N.Z. (SBS)	79.45 (4.96)	76.53 (6.85)	78.35 (6.29)	78.45 (7.21)	77.15 (6.8)	77.04 (6.89)	86.83 (5.03)	79.11 (6.29)
N.Z. (BTB)	66.95 (5.04)	74.16 (6.98)	80.34 (5.98)	86.52 (5.97)	67.98 (5.71)	74.16 (5.79)	78.28 (6.63)	75.48 (6.01)
N.Z. (DM)	77.84 (5.65)	77.03 (5.36)	77.2 (6.89)	77.35 (5.95)	77.32 (6.37)	77.69 (5.91)	87.26 (5.79)	78.81 (5.99)
SNG (NI)	75.02 (7.52)	74.95 (7.03)	73.37 (5.9)	71.96 (6.77)	73.75 (6.64)	72.48 (7.5)	88.17 (5.44)	75.67 (6.69)
SNG (SA)	72.1 (7.17)	74.33 (7.32)	74.06 (7.22)	76.08 (7.15)	72.51 (6.82)	75.12 (5.93)	87.28 (5.73)	75.93 (6.76)
SNG (ME)	75.33 (7.15)	75.81 (6.47)	75.67 (6.34)	75.26 (6.78)	71.89 (7.38)	73.58 (6.81)	85.76 (7.19)	76.19 (6.87)
SNG (SBS)	74.47 (7.8)	73.75 (6.76)	74.23 (7.13)	74.13 (8.26)	73.23 (6.18)	72.55 (6.87)	86.38 (7.37)	75.53 (7.2)
SNG (BTB)	66.95 (7.25)	74.16 (6.67)	69.01 (6.86)	84.46 (7.38)	76.22 (7.73)	76.22 (6.96)	81.37 (6.27)	75.48 (7.02)
SNG (DM)	73.33 (6.12)	74.51 (6.19)	74.59 (6.86)	73.95 (7.26)	73.31 (6.87)	73.41 (7.18)	87.24 (6.13)	75.76 (6.66)
A2. Mean (SD) Emotional familiarity								
Britain (NI)*	6.66 (1.24)	6.69 (1.2)	6.86 (1.08)	6.86 (1.14)	6.45 (1.09)	6.62 (1.28)	6.42 (.84)	6.65 (1.12)
Britain (SA)*	6.52 (.96)	6.62 (1.25)	6.76 (1.33)	6.86 (1.12)	7.1 (.96)	6.45 (1.21)	6.66 (1.18)	6.71 (1.14)
Britain (ME)*	6.63 (1.14)	6.89 (1.12)	6.55 (1.12)	7.07 (1.2)	6.56 (1.16)	6.83 (1.31)	6.35 (1.24)	6.7 (1.18)
Britain (SBS)*	6.83 (1.17)	6.66 (1.18)	6.56 (1.33)	6.73 (1.12)	6.69 (1.28)	6.18 (1.01)	7.1 (1.14)	6.68 (1.18)
Britain (BTB)*	6.69 (1.14)	6.52 (.9)	6.31 (1.05)	7 (1.23)	7.07 (1.16)	6.93 (1.17)	6.65 (1.05)	6.74 (1.1)
Britain (DM)*	6.83 (1.23)	6.18 (1.15)	6.69 (1.11)	6.69 (1.02)	6.28 (1.22)	6.52 (1.15)	6.79 (1.24)	6.57 (1.16)
Chile (NI)	5.57 (1.16)	5.73 (1.18)	5.5 (.99)	5.87 (.99)	5.5 (1.07)	5.6 (1.15)	5.66 (1.24)	5.63 (1.11)
Chile (SA)	5.6 (1.13)	5.66 (1.19)	5.36 (1.09)	5.91 (1.21)	5.47 (1.12)	5.4 (1.09)	5.63 (1.31)	5.58 (1.16)
Chile (ME)	5.67 (1.16)	5.56 (1.23)	5.6 (1.29)	5.5 (1)	5.6 (1.23)	5.29 (.96)	5.33 (1.19)	5.51 (1.15)
Chile (SBS)	5.29 (.99)	5.73 (1.15)	5.67 (1.23)	5.87 (1.1)	5.4 (1.22)	5.59 (1.26)	5.43 (1.18)	5.57 (1.16)
Chile (BTB)	5.26 (1.16)	5.94 (1.05)	5.6 (1.01)	5.63 (1.13)	5.57 (1.3)	5.43 (1.14)	5.74 (1.07)	5.6 (1.12)
Chile (DM)	5.54 (1.18)	5.53 (1.08)	5.4 (1.11)	5.74 (1.07)	5.88 (1.21)	5.54 (1.24)	5.56 (1.05)	5.6 (1.13)
N.Z. (NI)	5.6 (1.1)	5.84 (1.3)	5.49 (1.23)	5.19 (1.04)	5.46 (1.11)	5.22 (1.08)	5.4 (1.21)	5.46 (1.15)
N.Z. (SA)	5.98 (1.22)	5.57 (1.24)	5.91 (1.09)	5.64 (1.12)	5.74 (1.19)	5.43 (1.28)	5.8 (1.03)	5.72 (1.17)
N.Z. (ME)	5.87 (1.18)	5.8 (1.19)	5.77 (1.12)	5.53 (1.16)	5.77 (1.14)	5.94 (1.2)	5.77 (.99)	5.78 (1.14)
N.Z. (SBS)	6.14 (.96)	5.5 (1.15)	5.84 (1.22)	5.46 (1.23)	5.57 (1.04)	5.57 (1.03)	6.01 (1.01)	5.73 (1.09)
N.Z. (BTB)	5.57 (1.1)	5.73 (1.24)	5.6 (1.03)	5.29 (1.18)	6.01 (1.09)	5.67 (1.19)	5.46 (1.16)	5.62 (1.14)
N.Z. (DM)	5.63 (1.01)	5.9 (1.18)	5.57 (1.13)	5.97 (1.04)	6.15 (1.03)	5.91 (1.28)	5.87 (1.18)	5.86 (1.12)
SNG (NI)	4.71 (1.12)	4.30 (1.23)	4.51 (1.29)	4.58 (1.18)	4.92 (1.25)	4.2 (.96)	4.2 (1.02)	4.49 (1.15)
SNG (SA)	4.30 (1.1)	4.65 (1.11)	4.88 (1.1)	4.58 (1.17)	4.23 (1.02)	4.78 (1.17)	4.23 (1.11)	4.52 (1.11)
SNG (ME)	4.47 (.98)	4.99 (1.07)	4.68 (1.18)	4.82 (1.12)	4.78 (1.09)	4.41 (1.12)	4.78 (1.2)	4.7 (1.11)
SNG (SBS)	4.95 (1.02)	4.71 (1.16)	4.41 (1.1)	5.09 (1.23)	4.51 (1.13)	4.44 (1.27)	4.61 (1.03)	4.67 (1.13)
SNG (BTB)	4.44 (1.06)	4.68 (1.12)	4.82 (1.3)	4.51 (1.29)	4.37 (1.21)	4.74 (1.23)	4.71 (1.23)	4.61 (1.21)
SNG (DM)	4.61 (1.21)	4.75 (1.22)	5.05 (1.11)	4.58 (1.2)	4.48 (1.07)	4.23 (1.25)	4.31 (1.26)	4.57 (1.19)

This table includes mean and standard deviation for each Country of Origin of the presented actors and each emotional expression for emotional recognition (%) and racial and emotional familiarity (Likert Scale: 1–9). Abbreviations stand for No Intervention (NI), Shape Assessment (SA), Mere Exposure (ME), Side-by-Side (SBS), Back-to-Back (BTB) and Dynamic Morphing (DM). Asterisks signify categories higher than their respective and other non-British actor conditions at *p* < .001. These findings support the notion of the own-culture emotional recognition advantage (Elfenbein & Ambady, 2002a, 2002b; 2003; Elfenbein et al., 2007; Elfenbein, 2013, 2015).

To explore emotional familiarity outcomes, an analysis of variance with Independent Variables Cohort Group (No Intervention vs. Shape Assessment vs. Mere Exposure vs. Side-by-Side vs. Back-to-Back vs. Dynamic Morphing) and Actor Origin (Britain vs. Chile vs. New Zealand vs. Singapore) and Facial Emotion (Anger vs. Fear vs. Disgust vs. Sadness vs. Surprise vs. Happiness vs. Neutral) was run. The resulting model provided evidence for significant differences for Actor Origin (F (3, 87) = 547.69; *p* < .001; η^2^_p _= .95; SE = .03; *B* = +∞). Bonferroni-corrected comparisons showed that British expressions were significantly more emotionally familiar than Chilean (*p* < .001; *d* = .97), New Zealand (*p* < .001; *d* = .88) and Singaporean expressions (*p* < .001; *d* = 1.84; see Table 2). No other significant effects were reported including non-significance for emotional familiarity differences between the six Cohort Groups and Bayesian evidence for proximate scores between the six Cohort Groups (F (5, 145) = .68; *p* = .91; η^2^_p _= .01; SE = .09; *B* = .11). This finding suggested that between Cohort Group emotional familiarity differences would not bias emotional familiarity comparisons in subsequent phases. Finally, it is noteworthy and very critical for the current outcomes that an analysis of variance for familiarity ratings for racial characteristics revealed that there were no significant differences and strong Bayesian evidence for proximate ratings between actors’ (F (5, 145) = .33; *p* = .9; η^2^_p _= .01; SE = .06; *B* = .09). No other significant effects of interactions were revealed for racial familiarity. These findings suggested that differences in the perception of racial characteristics would not impact the current and subsequent findings. No significant differences for gender were reported during this phase.

### Results: Phase Two

**Figure 2.**
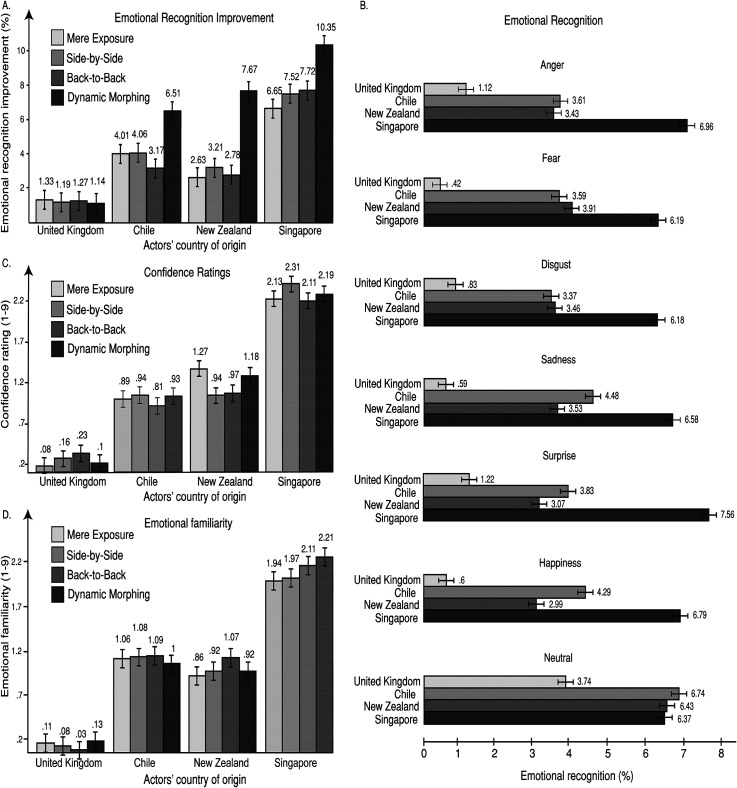
Graphical illustrations showing all significant interactions among Cohort, Confidence, Actor Origin, and Emotion for phase three for the entire populations sample (*n* = 180); (A) Emotional recognition improvement. (B) Emotional recognition. (C) Confidence ratings. (D) Emotional familiarity. Our findings show comparisons only between exploratory methods because the latter were in all respects higher than No Intervention and Shape Assessment. The findings of phase three and the currently illustrated findings strongly support that the best method for the improvement of cross-cultural emotional dialects was Dynamic Morphing. Bars indicate ±2 standard errors of the mean.

To explore the emotional recognition outcomes in phase two, an analysis of variance with Independent Variables Cohort Group (Mere Exposure vs. Side-by-Side vs. Back-to-Back vs. Dynamic Morphing) and Actor Origin (Britain vs. Chile vs. New Zealand vs. Singapore) and Facial Emotion (Anger vs. Fear vs. Disgust vs. Sadness vs. Surprise vs. Happiness vs. Neutral) was run. The resulting model provided evidence for significant differences for Actor Origin (F (3, 87) = 900.49; *p* < .001; η^2^_p _= .97; SE = .25; B = +∞). Bonferroni-corrected comparisons showed that British expressions were recognised significantly better than Chilean (*p* < .001; *d* = 1.63), New Zealand (*p* < .001; *d* = 1.65) and Singaporean expressions (*p* < .001; *d* = 2.15; see Figure 2). These results provided support for the suggestion that there is an own-culture emotional recognition advantage (Elfenbein & Ambady, 2002a). The analyses did not reveal any further significant outcomes or interactions. Critically, no significant effects were reported for emotional recognition differences between the four Cohort Groups and a trend for Bayesian evidence for proximate emotional recognition scores was revealed between the four Cohort Groups (F (3, 87) = 1.07; *p* = .39; η^2^_p _= .03; SE = .31; *B* = .41; see Figure 2). Similar outcomes were reported for response confidence for emotional recognition. An important effect of Actor Origin was reported (F (3, 87) = 193.08; *p* < .001; η^2^_p _= .87; SE = .42; *B* = +∞). Bonferroni-corrected comparisons revealed that British expressions were rated with significantly higher confidence for emotional recognition than Chilean (*p* < .001; *d* = .63), New Zealand (*p* < .001; *d* = .76) and Singaporean expressions (*p* < .001; *d* = 1.24). No other comparisons or interaction were significant for response confidence for emotional recognition (see Figure 2).

To explore emotional familiarity outcomes, an analysis of variance with Independent Variables Cohort Group (Mere Exposure vs. Side-by-Side vs. Back-to-Back vs. Dynamic Morphing) and Actor Origin (Britain vs. Chile vs. New Zealand vs. Singapore) and Facial Emotion (Anger vs. Fear vs. Disgust vs. Sadness vs. Surprise vs. Happiness vs. Neutral) was run. The resulting model provided evidence for significant differences for Actor Origin (F (3, 87) = 144.86; *p* < .001; η^2^_p _= .83; SE = .05; *B* = +∞). Bonferroni-corrected comparisons showed that British expressions were significantly more emotionally familiar than Chilean (*p* < .01; *d* = .35), New Zealand (*p* < .01; *d* = .34) and Singaporean expressions (*p* < .01; *d* = .39; see Figure 2). We critically reported non-significance for emotional familiarity differences between the four Cohort Groups and strong Bayesian evidence for proximate scores between the four Cohort Groups (F (3, 87) = .06; *p* = .98; η^2^_p _< .001; SE = .09; *B* = .05). Finally, an analysis of variance for familiarity ratings for racial characteristics revealed that there were no significant differences and a trend for Bayesian evidence for proximate ratings between actors’ countries of origin (F (3, 87) = 1.06; *p* = .37; η^2^_p _= .03; SE = .05; *B* = .45). No other significant effects or interactions were revealed for racial familiarity (see Figure 2). No significant differences for gender were reported during this phase; significant differences were not found for 1st or 2nd face order emotional and racial familiarity ratings during this phase.

Interestingly, analyses of variance comparisons overall between phase one and phase two for the cohorts that engaged in emotional recognition during phase two (Mere Exposure, Side-by-Side, Back-to-Back and Dynamic Morphing) revealed that emotional recognition (F (1, 29) = .53; *p* = .66; η^2^_p _= .01; SE = .21; *B* = .32), confidence for emotional recognition (F (1, 29) = 1.87; *p* = .31; η^2^_p _= .02; SE = .47; *B* = .41), emotional familiarity (F (1, 29) = .49; *p* = .96; η^2^_p _< .01; SE = .52; *B* = .09) and racial familiarity (F (1, 29) = 1.02; *p* = .41; η^2^_p _= .02; SE = .35; *B* = .39) were not significantly different and provided Bayesian evidence for proximate ratings between the two phases. It is particularly interesting that this effect took place between two phases with distinctly different tasks (see Figure 1: A1–4). This finding could suggest that non-interactive exposure to cross-cultural faces could not be sufficient for emotional recognition improvements. No further effects or interactions were reported for these analyses.

### Results: Phase Three

To explore participant improvements in cross-cultural emotional recognition, we initially deduced the final ratings for all variables in phase three from the baseline scores in phase one: X=PhaseThreeRatings−PhaseOneBaseline . Then we conducted a series of analyses of variance. For emotional recognition improvements (%), an analysis of variance with Independent Variables Cohort Group (No Intervention vs. Shape Assessment vs. Mere Exposure vs. Side-by-Side vs. Back-to-Back vs. Dynamic Morphing) and Actor Origin (Britain vs. Chile vs. New Zealand vs. Singapore) and Facial Emotion (Anger vs. Fear vs. Disgust vs. Sadness vs. Surprise vs. Happiness vs. Neutral) was run. The resulting model showed that there were significant differences between Cohort Groups (F (5, 145) = 157.71; *p* < .001; η^2^_p_ = .85; SE = .25; *B* = +∞). Dynamic Morphing was higher for emotional recognition improvements than Back-to-Back (*p* < .001; *d* = .39), Side-by-Side (*p* < .001; *d* = .35), Mere Exposure (*p* < .001; *d* = .41), Shape Assessment (*p* < .001; *d* = .61) and No Intervention (*p* < .001; *d* = 1.45; see Figure 2). Back-to-back was also higher than Shape Assessment (*p* < .01; *d* = .21) and No Intervention (*p* < 001; *d* = .62). The same effect was observed for Side-by-Side compared to Shape Assessment (*p* < .01; *d* = .25) and No Intervention (*p* < .001; *d* = .69). Mere exposure was also higher than Shape Assessment (*p* < .01; *d* = .19) and No Intervention (*p* < .001; *d* = .47). These findings suggest that explorative methods led to higher emotional recognition improvements compared to control conditions and that morphing was the most efficient method for the improvement of emotional recognition (see Figure 2).

The ANOVA model for emotional recognition improvements also revealed significant effects for Actor Origin (F (3, 87) = 195.93; *p* < .001; η^2^_p_ = .82; SE = .22; *B* = +∞). Interestingly, Singaporean actors showed the highest improvement for emotional recognition compared to British (*p* < .001; *d* = .47), Chilean (*p* < .001; *d* = .39) and New Zealand actors (*p* < .001; *d* = .54). Chilean actors showed higher emotional recognition improvement than British actors (*p* < .001; *d* = .59) and the same effect was revealed for New Zealand actors compared to British actors (*p* < .001; *d* = .53; see Figure 2). These interesting effects suggest that explorative interactive methods for the improvement of cross-cultural emotional recognition had the highest effects for other-culture expressions and particularly Singaporean actors, but they did not have an effect for own-culture emotional expressions (Chiao et al., 2008; Liu et al., 2015; Wheeler et al., 2011).

The ANOVA for emotional recognition improvements also revealed an effect of Emotion (F (6, 174) = 195.93; *p* < .001; η^2^_p_ = .82; SE = .22; *B* = +∞). Interestingly, the only significant effects of this analysis was that Neutral Faces were significantly reduced from their baseline scores compared to Angry (*p* < .001; *d* = 1.55), Fearful (*p* < .001; *d* = 1.54), Disgusted (*p* < .001; *d* = 1.33), Sad (*p* < .001; *d* = 1.56). Happy (*p* < .001; *d* = 1.59) and Surprised Faces (*p* < .001; *d* = 1.18; see Figure 2). This effect could suggest that participants were able to interpret emotional expressions much more accurately in phase three than in phase one resulting in possibly less misclassifications of emotional faces as neutral (see Bjornsdottir & Rule, 2021; Tae et al., 2020; Zebrowitz, 2017; Zebrowitz et al., 2012). Two highly significant interactions for Cohort by Actor Origin (F (15, 435) = 18.86; *p* < .001; η^2^_p_ = .39; SE = .62; *B* = +∞) and Actor Origin by Emotion effects (F (18, 522) = 10.01; *p* < .001; η^2^_p_ = .51; SE = .41; *B* = +∞) were also revealed (see Figure 2).

A similar pattern of results was observed for improvements in confidence of emotional recognition. A significant Cohort effect was revealed (F (5, 145) = 158.22; *p* < .001; η^2^_p_ = .85; SE = .05; *B* = +∞). Dynamic Morphing was higher for confidence improvements in emotional recognition than Shape Assessment (*p* < .001; *d* = .62) and No Intervention (*p* < .001; *d* = 1.14). Similar effects were observed for Actor Origin (F (3, 87) = 221.29; *p* < .001; η^2^_p_ = .88; SE = .04; *B* = +∞). In these comparisons again Singaporean actors showed more improvement than Chilean (*p* < .001; *d* = .76), New Zealand (*p* < .001; *d* = .66) and British actors (*p* < .001; *d* = 1.08). Chilean actors were higher than British actors (*p* < .01; *d* = .28). The same pattern was observed for New Zealand actors versus British actors (*p* < .001; *d* = .38). A Cohort by Actor Origin interaction was revealed for confidence for emotional recognition (F (15, 435) = 16.97; *p* < .001; η^2^_p_ = 37; SE = .12; *B* = +∞; see Figure 2).

Similar results were observed for emotional familiarity improvements. A significant Cohort effect was revealed (F (5, 145) = 85.33; *p* < .001; η^2^_p_ = .75; SE = .05; *B* = +∞). Dynamic Morphing was higher for ratings for emotional familiarity improvements than Shape Assessment (*p* < .001; *d* = .59) and No Intervention *p* < .001; *d* = .38). Similar effects were observed for Actor Origin (F (3, 87) = 184.07; *p* < .001; η^2^_p_ = .86; SE = .04; *B* = +∞). In these comparisons, Singaporean actors showed more improvement than British actors only (*p* < .001; *d* = .97). Chilean actors were higher than British actors (*p* < .01; *d* = .28). The same pattern was observed for New Zealand actors versus British actors (*p* < .001; *d* = 1.17). A Cohort by Actor Origin interaction was revealed for improvements for emotional familiarity (F (15, 435) = 14.67; *p* < .001; η^2^_p_ = 34; SE = .12; *B* = +∞; see Figure 2). No significant differences were reported for improvements in racial familiarity and Bayesian evidence for equivalence of significance were reported for Independent Variables Cohort (F (5, 145) = .46; *p* = .81; η^2^_p_ = .01; SE = .05; *B* = .17) and Actor Origin (F (5, 145) = .88; *p* = .45; η^2^_p_ = .02; SE = .04; *B* = .21). No other effects or interactions were significant for these analyses. No gender effects were observed during this analysis. An important outcome for these analyses was that the measures of dispersion were very high. This unexpected outcome could be due to the large differences between the high improvement ratings in response to Singaporean actors compared to Chilean and New Zealand actors and critically the null and negative improvement ratings in response to British actors (see Figure 2).

## Experimental Stage Two

### Aims

The main aim of this stage was to further explore dynamic morphing as an emotional dialect recognition method and determine in what format it is most effective for improving cross-cultural emotional recognition. Further aims of the current stage involved testing whether we could provide yet another replication of the own-culture emotional recognition advantage including confidence for own-culture emotional recognition and emotional familiarity.

### Participants

**Figure 3.**
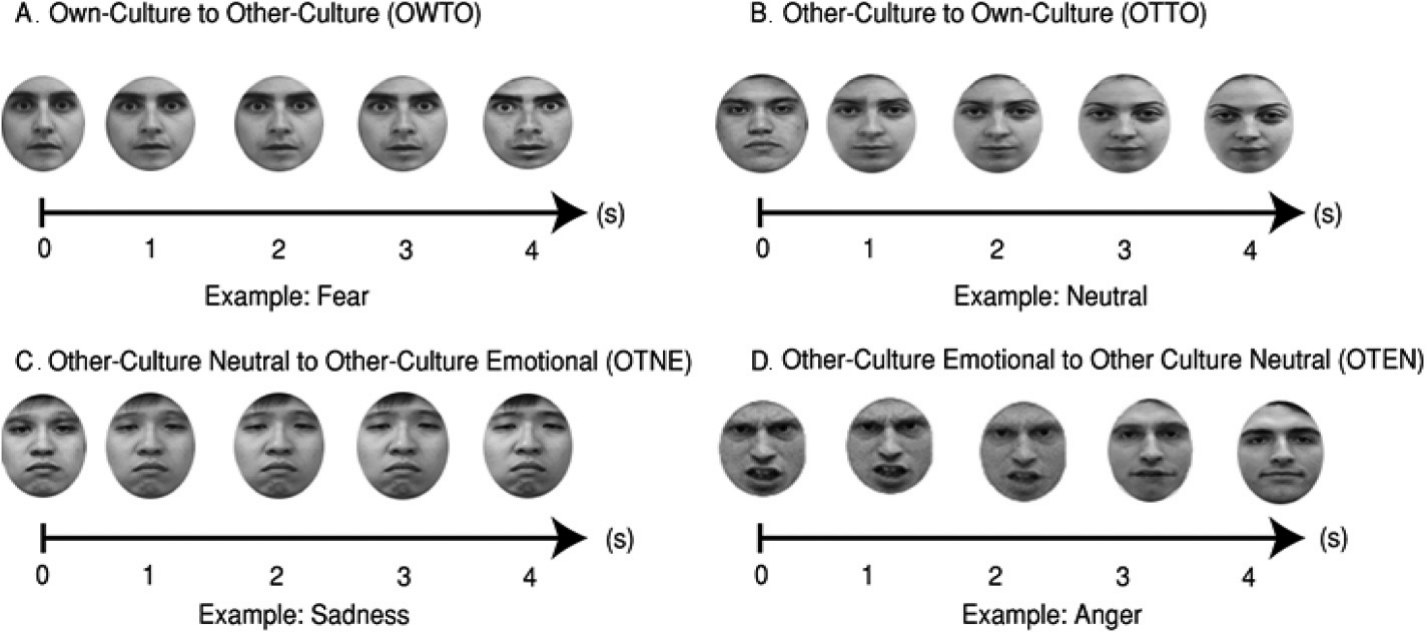
Morphing sequences for Stage Two, phase two including (A) own-to-other culture, (B) other-to-own culture, (C) other-culture neutral to other-culture emotional, and (D) other-culture emotional to other-culture neutral facial morphing. A fixation cross for 1 s (see Figure 1(D)) was presented before every trial in every condition. The engagement tasks included in each condition can be seen in Figure 1. Further examples and illustrations of these morphing methods can be found in https://osf.io/3z97s/ and https://osf.io/cdvhz/ and https://osf.io/syvf9/.

A power calculation based on medium sizes indicated that 120 participants were required for this stage (*P*_(1−β)_ ≥ .9; *p* ≤ .05; η^2^_p _≥ .06; Faul et al., 2009; *P* (H_0_) ≥ .9; *B* < .33; η^2^_p_ [0, < .001]; Kelter, 2021). A total of 131 participants (65 females) volunteered to participate in this study. All participants reported normal or corrected-to-normal vision. The inclusion criteria and screening assessments were the same as Stage One. Data from six participants were excluded because they failed to attend the second and third phases of the experiment. Data from three participants were excluded due to SPRHQ scores that indicated a possible psychiatric diagnosis. Data from one participant were excluded due to scores that indicated possible traits for alexithymia. Data from one participant were excluded due to having a joint nationality. The final sample consisted of 120 participants (60 females) with mean age 27.48 years (SD = 2.71; see Table 3). The participants were randomly allocated in four cohorts depending on the type of dynamic morphing that was used during phase two (see Figure 3). All participants gave informed consent to participate in this study and for their data to be used for further research purposes. The experiment was approved by the School of Psychology, University of Nottingham.

**Table 3.** Participant characteristics for experimental stage two.

Method of dynamic morphing	*n* (female)	Age mean (SD)	CDQ mean (SD)					ERQ mean (SD)	
			PD	IND	MAS	U-A	LTO	CR	ES
Own-to-other culture	30 (15)	27.08 (2.51)	45.13 (3.5)	62.13 (3.08)	44.97 (2.98)	68.27 (2.96)	53.26 (1.55)	24.17 (2.56)	8.27 (.78)
Other-to-own culture	30 (15)	28.14 (3.02)	45.1 (2.98)	62.23 (3.93)	45.44 (3.1)	67.8 (3.31)	52.09 (2.02)	24.97 (2.2)	7.93 (.87)
Neutral-to- emotional (OC)	30 (15)	27.57 (2.5)	45.87 (2.89)	62.2 (3.33)	44.43 (3.33)	67.71 (3.64)	52.8 (2.1)	25.15 (2.64)	7.95 (.89)
Emotional-to- neutral (OC)	30 (15)	27.13 (2.79)	45.5 (2.78)	63.24 (2.97)	44.4 (2.97)	67.74 (3.3)	53.3 (1.75)	24.11 (2.47)	8.03 (.85)
Bayes factor, ANOVA and effect sizes for each category									
p-value (B factor)			.75 (.45)	.54 (.98)	.59 (1.04)	.55 (.96)	.67 (.53)	.42 (2.94)	.43 (2.91)
η^2^_p_			.01	.02	.02	.01	.01	.03	.03

Participant characteristics for Stage Two for each cohort including own-to-other, other-to-own, other-culture neutral-to-emotional and other culture emotional-to-neutral. OC stands for other culture in the last two cohorts. Age, gender, Hofstede Cultural Dimensions Questionnaire (CDQ) with scores for power distance (PD), individualism (IND), masculinity (MAS), uncertainty-avoidance (U-A) and long-term orientation and emotional regulation questionnaire (ERQ) with scores for cognitive re-appraisal (CR) and emotional suppression (ES) per cohort are included. In the bottom part of the *p*-values, B factor values and partial eta-squared values for every analysis. See also https://osf.io/3z97s/ and https://osf.io/cdvhz/ and https://osf.io/syvf9/.

### Procedure

The same stimuli including image coding and processing procedures used in Stage One were used for Stage Two (see *Stage One: Procedures*). For phases one and three in Stage Two, the experimental sequence and procedures were also identical to Stage One (see *Procedure: Phase One* and *Phase Two* in Stage One; see also Figures 1 and 2). Phase two was different for experimental Stage Two.

### Procedure: Phase Two

Phase two took place one week after phase one on the same day and timeslot as phase one. It included four between-subjects conditions. Thirty participants were randomly allocated in each condition (see Table 1). The conditions included only dynamic morphing (**DM**). The refresh rate for dynamic morphing was set at 60 Hz (16.67 ms) and the resolution of the morphed images was set at 1024 × 768 pixels using Abrosoft Fantamorph Pro (for further morphing illustrations, parameters and code, see https://osf.io/syvf9/). The conditions were morphing from own-culture to other-culture faces (**OWTO**), other-culture to own-culture faces (**OTTO**), other-culture neutral to other-culture emotional faces (**OTNE**) and other-culture emotional faces to other-culture neutral faces (**OTEN**).

In the **OWTO** condition, the experiment started with a fixation cross for 1 s. After the fixation cross, an own-culture face was morphed into an other-culture face. The two faces always expressed the same freely-expressed emotion. The morphing lasted for 4 s. The faces included 105 own-culture faces, 35 Chilean, 35 New Zealand and 35 Singaporean faces presented with order randomised. The other-culture faces included five angry, disgusted, fearful, sad, surprised, happy or neutral expressions from each culture. Thirty-five pairs of pattern blurs were also presented in morph as catch trials (*n*_trials _= 245). After the presentation a blank screen was shown for 1 s. Participants were then asked to answer what emotion the faces were expressing, including “other,” “different” and “non-facial.” Participants used the mouse to make their recognition selection from nine textboxes with order of positioning randomised in each trial. This question was always followed by a Likert scale on how confident they were for their choice ranging from 1 (not at all) to 5 (moderately) to 9 (very). Participants were also asked to rate in pseudorandomised order (resulting in 50% responses for) either the first or second face for emotional familiarity and racial familiarity from 1 (not at all) to 5 (moderately) to 9 (very). After this task, we used conditional branching. In the case of a correct recognition answer, an on-screen message stated “Congratulations, your recognition answer was correct” or, in the case of an incorrect answer, “I am sorry, your recognition answer was not correct” for 2 s. Then an on-screen message stated “The correct answer was x” for 2 s (e.g., fear or anger or disgust) and the morphing sequence was shown again shown for 4 s. A blank screen for 1 s was presented before the next trial. No actor identity was repeated during this condition within or between trials. The gender of the presented actors was randomised for own and other-culture faces in all conditions (see Figure 3).

In the **OTTO** condition, the experiment started with a fixation cross for 1 s. After the fixation cross, an other-culture face was morphed into an own-culture face. The two faces always expressed the same freely-expressed emotion. The morphing lasted for 4 s. The faces included 105 other-culture and 105 own-culture faces. The own-culture faces included 15 angry, disgusted, fearful, sad, surprised, happy, or neutral expressions and the other-culture faces included five angry, disgusted, fearful, sad, surprised, happy or neutral expressions. Thirty-five pairs of pattern blurs were also presented in morph as catch trials (*n*_trials _= 245). The engagement tasks, interactive feedback and experimental restrictions were identical with the **OWTO** condition (see Figure 3).

In the **OTNE** condition, the experiment started with a fixation cross for 1 s. After the fixation cross, a neutral other-culture face was morphed into an emotional other-culture face. The morphing lasted for 4 s. The faces included 105 other-culture neutral faces and 105 emotional other-culture faces. The emotional other-culture faces included 15 angry, disgusted, fearful, sad, surprised, happy or neutral expressions. Thirty-five pairs of pattern blurs were also presented in morph as catch trials (*n*_trials _= 245). The engagement tasks and interactive feedback were identical with the **OWTO** condition (see Figure 3).

In the **OTEN** condition, the experiment started with a fixation cross for 1 s. After the fixation cross, an emotional other-culture face was morphed into a neutral other-culture face. The morphing lasted for 4 s. The faces included 105 other-culture emotional faces and 105 neutral other-culture faces. The emotional other-culture faces included 15 angry, disgusted, fearful, sad, surprised, happy or neutral expressions. Thirty-five pairs of pattern blurs were also presented in morph as catch trials (*n*_trials _= 245). The engagement tasks and interactive feedback were identical with the **OWTO** condition (see Figure 3).

### Results: Stage Two: Phase One

**Figure 4.**
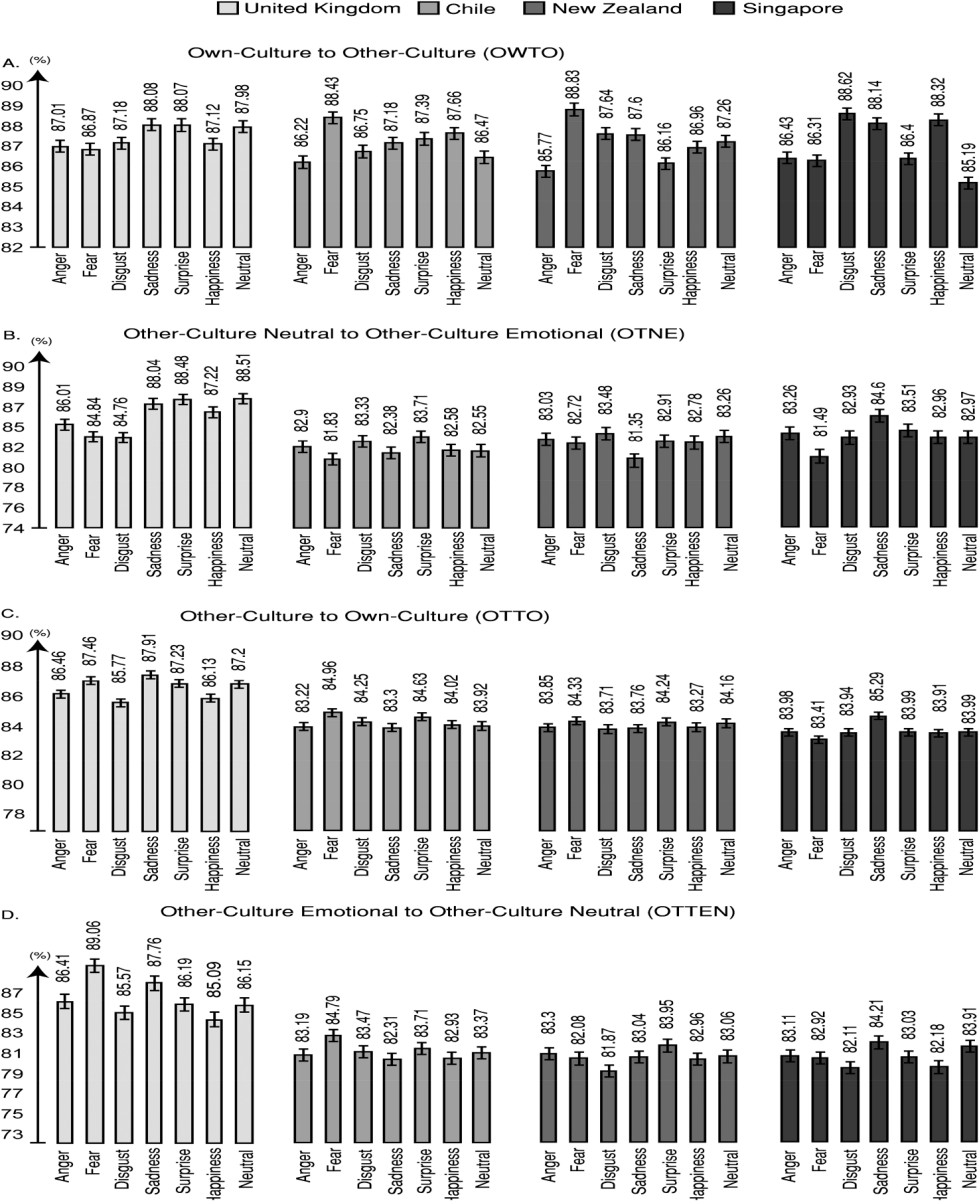
Percentage performance for each condition (A) OWTO, (B) OTTO, (C) OTNE, and (D) OTEN; for Stage Two, phase three. The OWTO condition was the only condition that provided Bayesian evidence for significance of equivalence between British, Chilean, New Zealand and Singaporean actors. This finding suggests that own-to-other culture morphing induced sufficiently high levels of emotional recognition improvement to level the own-culture recognition advantage observed in the current and previous research (Elfenbein & Ambady, 2002a, 2002b). Error bars indicate ±2 standard errors of the mean.

To explore the emotional recognition outcomes in Stage Two, phase one, an analysis of variance with Independent Variables Cohort Group (OWTO vs. OTTO vs. OTNE vs. OTEN) and Actor Origin (Britain vs. Chile vs. New Zealand vs. Singapore) and Facial Emotion (Anger vs. Fear vs. Disgust vs. Sadness vs. Surprise vs. Happiness vs. Neutral) was run. The resulting model provided evidence for significant differences for Actor Origin (F (3, 87) = 897.43; *p* < .001; η^2^_p _= .98; SE = .16; *B* = +∞). Bonferroni-corrected comparisons showed that British expressions were recognised significantly better than Chilean (*p* < .001; *d* = 2.18), New Zealand (*p* < .001; *d* = 2.24) and Singaporean expressions (*p* < .001; *d* = 3.22). These results provided replicated support for the suggestion that there is an own-culture emotional recognition advantage (Elfenbein & Ambady, 2002a). The analyses also revealed significant differences for Emotion (F (6, 174) = 267.28; *p* < .001; η^2^_p _= .91; SE = .23; *B* = +∞) and a significant Facial Emotion to Actor Origin interaction (F (18, 522) = 91.53; *p* < .001; η^2^_p _= .76; SE = .31; *B* = +∞). Critically, no other significant effects were reported including non-significance for emotional recognition differences between the four Cohort Groups and Bayesian evidence for proximate emotional recognition scores between the Cohort Groups (F (3, 87) = .58; *p* = .89; η^2^_p _= .01; SE = .29; *B* = .12). Similar outcomes were reported for response confidence for emotional recognition. An important effect of Actor Origin was reported (F (3, 87) = 233.66; *p* < .001; η^2^_p _= .89; SE = .04; *B* = +∞). Bonferroni-corrected comparisons revealed that British expressions were rated with significantly higher confidence for emotional recognition than Chilean (*p* < .001; *d* = .85), New Zealand (*p* < .001; *d* = .82) and Singaporean expressions (*p* < .001; *d* = 1.4). No other comparisons or interaction were significant for response confidence for emotional recognition (see Figure 4).

To explore emotional familiarity outcomes, an analysis of variance with Independent Variables Cohort Group (OWTO vs. OTTO vs. OTNE vs. OTEN) and Actor Origin (Britain vs. Chile vs. New Zealand vs. Singapore) and Facial Emotion (Anger vs. Fear vs. Disgust vs. Sadness vs. Surprise vs. Happiness vs. Neutral) was run. The resulting model provided evidence for significant differences for Actor Origin (F (3, 87) = 135.47; *p* < .001; η^2^_p _= .82; SE = .04; *B* = +∞). Bonferroni-corrected comparisons showed that British expressions were significantly more emotionally familiar than Chilean (*p* < .001; *d* = .97), New Zealand (*p* < .001; *d* = .88) and Singaporean expressions (*p* < .001; *d* = 1.84). No other significant effects were reported including non-significance for emotional familiarity differences between the Cohort Groups and Bayesian evidence for proximate scores between the Cohort Groups (F (3, 87) = .03; *p* = .99; η^2^_p _= .01; SE = .05; *B* = .06). An analysis of variance for familiarity ratings for racial characteristics revealed that there were no significant differences and a trend for Bayesian evidence for proximate ratings between actors’ countries of origin (F (3, 87) = 1.03; *p* = .348; η^2^_p _= .03; SE = .03; *B* = .46). No other significant effects of interactions were revealed for racial familiarity. No significant differences for gender were reported (see Figure 4).

### Results: Stage Two: Phase Two

To explore the emotional recognition outcomes in phase two, an analysis of variance with Independent Variables Cohort Group (OWTO vs. OTTO vs. OTNE vs. OTEN) and Actor Origin (Britain vs. Chile vs. New Zealand vs. Singapore) and Facial Emotion (Anger vs. Fear vs. Disgust vs. Sadness vs. Surprise vs. Happiness vs. Neutral) was run. The resulting model provided evidence for significant differences for Actor Origin (F (3, 87) = 678.19; *p* < .001; η^2^_p _= .96; SE = .24; *B* = +∞). Bonferroni-corrected comparisons showed that British expressions were recognised significantly better than Chilean (*p* < .001; *d* = 1.86), New Zealand (*p* < .001; *d* = 1.79) and Singaporean expressions (*p* < .001; *d* = 2.71). The analyses did not reveal any further significant outcomes or interaction. Critically, no significant effects were reported for emotional recognition differences between the Cohort Groups and Bayesian evidence for proximate emotional recognition scores were revealed between the Cohort Groups (F (3, 87) = .79; *p* = .51; η^2^_p _= .02; SE = .27; *B* = .29; see Figure 4). Similar outcomes were reported for response confidence for emotional recognition. An important effect of Actor Origin was reported (F (3, 87) = 291.29; *p* < .001; η^2^_p _= .91; SE = .04; *B* = +∞). Bonferroni-corrected comparisons revealed that British expressions were rated with significantly higher confidence for emotional recognition than Chilean (*p* < .001; *d* = .63), New Zealand (*p* < .001; *d* = .76) and Singaporean expressions (*p* < .001; *d* = 1.24). No other comparisons or interaction were significant for response confidence for emotional recognition (see Figure 4).

To explore emotional familiarity outcomes, an analysis of variance with Independent Variables Cohort Group (OWTO vs. OTTO vs. OTNE vs. OTEN) and Actor Origin (Britain vs. Chile vs. New Zealand vs. Singapore) and Facial Emotion (Anger vs. Fear vs. Disgust vs. Sadness vs. Surprise vs. Happiness vs. Neutral) was run. The resulting model provided evidence for significant differences for Actor Origin (F (3, 87) = 348.91; *p* < .001; η^2^_p _= .91; SE = .03; *B* = +∞). Bonferroni-corrected comparisons showed that British expressions were significantly more emotionally familiar than Chilean (*p* < .01; *d* = .38), New Zealand (*p* < .001; *d* = .44) and Singaporean expressions (*p* < .001; *d* = .53). No other significant effects were reported including none for emotional familiarity differences between the Cohort Groups and Bayesian evidence for proximate scores between the four Cohort Groups were reported (F (3, 87) = .41; *p* = .75; η^2^_p _< .001; SE = .03; *B* = .11). There were no significant differences and Bayesian evidence for proximate ratings between actors’ countries of origin (F (3, 87) = .49; *p* = .71; η^2^_p _< .001; SE = .07; *B* = .13). No other significant effects or interactions were revealed for racial familiarity. No significant differences for gender were reported. Analyses of variance comparisons overall between phase one and phase two revealed that emotional recognition (F (1, 29) = .51; *p* = .48; η^2^_p _= .01; SE = .72; *B* = .31), confidence for emotional recognition (F (1, 29) = 1.35; *p* = .25; η^2^_p _= .02; SE = .21; *B* = .49), emotional familiarity (F (1, 29) = .28; *p* = .61; η^2^_p _< .01; SE = .15; *B* = .17) and racial familiarity (F (1, 29) = .29; *p* = .59; η^2^_p _= .01; SE = .13; *B* = .21) were not significantly different and provided Bayesian evidence for proximate ratings between the two phases (see Figure 4).

### Results: Stage Two: Phase Three

For emotional recognition improvements (%), an analysis of variance with Independent Variables Cohort Group (OWTO vs. OTTO vs. OTNE vs. OTEN) and Actor Origin (Britain vs. Chile vs. New Zealand vs. Singapore) and Facial Emotion (Anger vs. Fear vs. Disgust vs. Sadness vs. Surprise vs. Happiness vs. Neutral) was run. The resulting model showed that there were significant differences between Cohort Groups (F (3, 87) = 55.53; *p* < .001; η^2^_p_ = .71; SE = .23; B = +∞). OWTO was higher for emotional recognition improvements than OTTO (*p* < .001; *d* = .65), OTNE (*p* < .001; *d* = .61) and OTEN (*p* < .001; *d* = .59). When we further examined these findings, an analysis of variance with Independent Variables Country of Origin (Britain vs. Chile vs. New Zealand vs. Singapore) and Emotion (Anger vs. Fear vs. Disgust vs. Sadness vs. Surprise vs. Happiness vs. Neutral) for each Cohort revealed that the only condition that provided Bayesian evidence for equivalence of significance testing for the likelihood of the data being observed under the null hypothesis between the scores for each country was OWTO (F (3, 87) = .18; *p* = .91; η^2^_p_ < .001; SE = .39; *B* = .09). These findings suggested that own-to-other culture dynamic morphing not only led to higher emotional recognition improvements compared to other morphing conditions but also that it was the only condition that provided sufficiently high emotional recognition improvements to allow us to report proximate scores between own-culture faces and Chilean, New Zealand and Singaporean faces (see Figure 4).

The ANOVA model for emotional recognition improvements also revealed significant effects for Actor Origin (F (3, 87) = 268.73; *p* < .001; η^2^_p_ = .92; SE = .31; *B* = +∞). Interestingly, like in Stage One, Singaporean actors showed the highest improvement for emotional recognition compared to British (*p* < .001; *d* = 1.92), Chilean (*p* < .001; *d* = .85) and New Zealand actors (*p* < .001; *d* = .84). Chilean actors were rated with higher emotional recognition than British actors (*p* < .001; *d* = 1.06) and the same effect was revealed for New Zealand actors compared to British actors (*p* < .001; *d* = 1.82). The ANOVA for emotional recognition improvements also revealed an effect of Emotion (F (6, 138) = 262.43; *p* < .001; η^2^_p_ = .92; SE = .29; *B* = +∞). Interestingly, the only significant effect of this analysis was that Neutral Faces were significantly reduced from their baseline scores compared to Angry (*p* < .001; *d* = 2.81), Fearful (*p* < .001; *d* = 2.42), Disgusted (*p* < .001; *d* = 2.32), Sad (*p* < .001; *d* = 1.56), Happy (*p* < .001; *d* = 2.27), Sad (*p* < .001; *d* = 2.23) and Surprised Faces (*p* < .001; *d* = 2.24). Significant interactions were also revealed for Cohort by Country of Origin (F (9, 207) = 4.61; *p* < .001; η^2^_p_ = .42; SE = .29; *B* = +∞) and Country of Origin by Emotion (F (18, 414) = 23.83; *p* < .001; η^2^_p_ = .57; SE = .29; B = +∞; see Figure 4). For improvements in confidence of emotional recognition only a significant Country of Origin effect was revealed (F (3, 87) = 120.33; *p* < .001; η^2^_p_ = .81; SE = .05; *B* = +∞). In these comparisons, Singaporean actors showed more improvement than Chilean (*p* < .01; *d* = .34), New Zealand (*p* < .001; *d* = .41) and British actors (*p* < .001; *d* = 1.15). The same pattern was observed for Chilean actors (*p* < .001; *d* = .82) and New Zealand actors (*p* < .001; *d* = .78) compared to British actors. For emotional familiarity improvements, only a significant Country of Origin effect was revealed (F (3, 87) = 55.13; *p* < .001; η^2^_p_ = .66; SE = .05; *B* = +∞). Singaporean actors were rated higher for emotional familiarity improvements than British (*p* < .001; *d* = .77), Chilean (*p* < .001; *d* = .42) and New Zealand Actors (*p* < .001; *d* = .45). Chilean (*p* < .001; *d* = .35) and New Zealand (*p* < .001; *d* = .33) also showed higher improvement than British participants. Racial familiarity ratings did not reveal significant differences and showed strong Bayesian evidence for proximate ratings (F (3, 87) = .06; *p* = .98; η^2^_p_ < .001; SE = .02; *B* = .07; see Figure 4).

## Summary of Findings

In the current research, we explored whether we could improve the recognition of cross-cultural facial-emotional expressions in British participants. We used a variety of methods to improve cross-cultural emotional recognition, such as different types of simultaneous own and other-culture presentations of facial expressions and dynamic morphing presentations of cross-cultural facial-emotional expressions. We found that training could improve the emotional recognition of other-culture emotional faces. Moreover, we showed that dynamic morphing from own-to-other-culture facial-emotional expressions provided emotional recognition improvements that allowed us to show Bayesian evidence for significance of equivalence for emotional recognition between own and other-culture emotional faces. We were able to provide two replications of the own-culture recognition advantage during the early phases of each stage of this research. We showed that the reported outcomes were due to increases in cross-cultural facial-emotional recognition performance and were not affected by ratings for racial familiarity.

## General Discussion

The psychological model of basic universal emotions suggests that certain facial-emotional expressions, such as anger, fear, disgust, happiness, sadness, surprise and neutral expressions, are a universal language of human communication. These emotions are expressed via facial expressions that include specific AUs, such as different mouth and eye movements. They can be recognised cross-culturally because they serve universal expression-related human needs (Keltner et al., 2019). One model in the area of cross-cultural emotional communication suggests that although universal emotions can in prototypical states, such as when expressing very intense and specific AUs, be a universal language of communication, different cultures also have specific dialects of emotion that are discrete and distinguishable. These dialects are more accurately recognised within rather than between cultures. This phenomenon is termed the own-culture emotional recognition advantage (see, e.g., Cowen et al., 2019; Crivelli & Fridlund, 2019; Laukka & Elfenbein, 2021). Our own research suggests that the own-culture emotional recognition advantage requires explicit awareness of a presented face (Tsikandilakis, Bali, et al., 2019; Tsikandilakis, Yu, et al. 2021). In this research project, we therefore implemented several explicit presentation methods to improve the cross-cultural recognition of emotion in British participants.

During the current studies, we provided an intense – and perhaps even overwhelming – plethora of findings. This was to some extent unavoidable given the exploratory nature of the current methodology that involved two experimental replications, eleven explorative experimental stages and an excess of 500 very strictly assessed and selected participants. We would like to instate for the reader the most critically important outcome of our research. The most critical of our findings was that we showed Bayesian evidence that own and other-culture emotional faces were processed with proximal emotional recognition accuracy. The method that allowed us to show such seminal results was dynamic morphing from own to other-culture emotional faces (**OWTO**). Despite the fact that almost all non-control methods we implemented led to increases in recognition performance, **OWTO** was the only condition that provided sufficiently high emotional recognition improvements to level the own-culture recognition advantage. This was potentially the most contributing for further research result of the current study (see e.g., Makowski, 2018; Premachandra & Lewis, 2022). This result was most likely due to the progressive and distinguishable transformation of known and well-understood own-culture facial-emotional characteristics to less familiar expressions of other-culture emotional expressions during own to other-culture dynamic morphing.

Diving deeper in the plethora of findings of the current research project, we observed several unexpected outcomes that were not part of the hypotheses of the current research. Firstly, own-culture faces did not show an improvement during any method we implemented. This effect was unanticipated but not necessarily inexplicable. It would be a scientific oxymoron to suggest that interactive feedback decreased the ability of the participants to perceive own-culture faces (Elfenbein, 2015). Instead, a more sensible explanation for this phenomenon is that participants dedicated a higher proportion of their attentional resources and cognitive reserve to novel stimuli, such as Chilean, New Zealand and Singaporean faces, resulting in a reduction in the recognition performance of learned stimulus types (Bak et al., 2016, 2019). A similar explanation could be extended to the unexpected outcome that Singaporean emotional faces showed the highest increase in recognition performance. This could be due to the expressive novelties of the specific emotional dialect. In our previous works (see Tsikandilakis, Kausel, et al., 2019; Tsikandilakis, Yu, et al. 2021), we showed that Singaporean dialects of facial emotion were evaluated as highly incomprehensible by British participants. It is possible that the novelty of these dialects attracted attentional resources and that they were the least likely stimulus type to be misperceived as a different emotional dialect, therefore, resulting in higher recognition improvements (see Inhelder et al., 2014; Mather, 2013; Ramírez Butavand et al., 2020).

Overall, the current results open pathways for further research and pose challenges in the novel topic of cross-cultural emotional-recognition performance improvements. They affirm the hypotheses of the current research, such as that contemporary – more visually nourishing and informative – methods, such as dynamic morphing, could provide the highest increases in recognition performance, that we could replicate the plethora of studies that showed an own-culture emotional recognition advantage and that the outcomes of the current study could be due to emotional recognition and not racial familiarity improvements (Brooks et al., 2019; Wong et al., 2020).

### Limitations

The current study included actors from Britain, Chile, New Zealand and Singapore and was conducted in Britain. The dataset was created during a previous research award by Universitas 21, and it was based on the collaborative institutional availability of the specific funding body and the funding reserves to conduct a single population study (see also Tsikandilakis, Kausel, et al., 2019). Further research could benefit from cross-cultural replications of the current findings (see Tsikandilakis, Yu, et al. 2021). We have provided strong evidence in this research project that cross-cultural emotional learning can be improved using interactive training. The current findings provide proof of concept that there is significant potential for the development of methods for improving cross-cultural emotional recognition (Makowski, 2018; Premachandra & Lewis, 2022). The creation of practical training methods for cross-cultural emotional communication can help us understand other cultures better. They can be a very valuable aid for people who are planning to travel, move, settle or simply come in contact with novel cultural groups and could constitute the next step of research stemming from the contributing findings of the current methodologically explorative paper (Berry, 1990, 2020; Hermans, 2020, 2001; Hermans & Kempen, 1998; Ringeval et al., 2019; Smith et al., 2013). In all the current experimental stages, British participants were selected with very strict criteria and provided evidence for no significant differences and Bayesian evidence for proximal scores in cultural and emotional questionnaires, proximate scores to our previous British population studies (see Tsikandilakis, Kausel, et al., 2019; pp. 14–15 Tsikandilakis, Yu, et al. 2021; pp. 9–12; Tsikandilakis, Leong, et al., 2021; pp. 10 & 30; see also Leong et al., 2023) and also, critically, no significant differences and Bayesian evidence for among cohorts and conditions expression recognition and emotional expression learning. Nevertheless, and despite that a national population is commonly inherently multi-culturally stratified, a significant unexplored avenue for further research (see Hall, 2022) and also possibly a bias (see Verkuyten, 2007) in relevant research is the assessment – or lack thereof – of within-population variances such racial background and culture of ancestry in exploring emotional recognition and emotional learning (see Tam & Milfont, 2020). Along these lines, in the introduction and discussion of the current manuscript we referred to “country” and “culture” as regards participants’ emotional dialects as they were quoted directly in the original sources we discoursed. In our own sections, we used the more neutral term “participants’ country of origin.” We consider the experimental avenue of examining diverse racial and cultural characteristics within a single country of origin a very important and contributing area for further experimental research and furthering our understanding of emotional dialects.

### Conclusions

In the current research project, we tested a variety of methods for improving cross-cultural facial-emotional recognition in a British population sample. We also tested whether we could replicate the own-culture facial-emotional recognition advantage and whether racial familiarity contributed to potential improvements in the recognition of emotion. We found that several methods led to recognition improvements. Dynamic masking from own to other-culture faces provided the highest improvement and Bayesian evidence for significance of equivalence for the recognition of own and other-culture emotional faces. We were able to provide two replications of the own-culture recognition advantage and provided evidence that improvements in the recognition of emotional dialects and not racial familiarity contributed to the outcomes of the current study. Overall, our findings suggested that interactive training enhanced the cross-cultural perception of emotion, and our outcomes could be evidence of the value and feasibility of developing educational methods for improving cross-cultural emotional communication.
